# Striatum-Mediated Deficits in Stimulus-Response Learning and Decision-Making in OCD

**DOI:** 10.3389/fpsyt.2020.00013

**Published:** 2020-02-05

**Authors:** Nole M. Hiebert, Marc R. Lawrence, Hooman Ganjavi, Mark Watling, Adrian M. Owen, Ken N. Seergobin, Penny A. MacDonald

**Affiliations:** ^1^ Schulich School of Medicine and Dentistry, University of Western Ontario, London, ON, Canada; ^2^ Brain and Mind Institute, University of Western Ontario, London, ON, Canada; ^3^ Department of Physiology and Pharmacology, University of Western Ontario, London, ON, Canada; ^4^ Department of Psychiatry, University of Western Ontario, London, ON, Canada; ^5^ Department of Psychology, University of Western Ontario, London, ON, Canada; ^6^ Department of Clinical Neurological Sciences, University of Western Ontario, London, ON, Canada

**Keywords:** obsessive compulsive disorder, neuroimaging, striatum, learning, decision-making

## Abstract

Obsessive compulsive disorder (OCD) is a prevalent psychiatric disorder characterized by obsessions and compulsions. Studies investigating symptomatology and cognitive deficits in OCD frequently implicate the striatum. The aim of this study was to explore striatum-mediated cognitive deficits in patients with OCD as they complete a stimulus-response learning task previously shown to differentially rely on the dorsal (DS) and ventral striatum (VS). We hypothesized that patients with OCD will show both impaired decision-making and learning, coupled with reduced task-relevant activity in DS and VS, respectively, compared to healthy controls. We found that patients with OCD (n = 14) exhibited decision-making deficits and learned associations slower compared to healthy age-matched controls (n = 16). Along with these behavioral deficits, OCD patients had reduced task-relevant activity in DS and VS, compared to controls. This study reveals that responses in DS and VS are altered in OCD, and sheds light on the cognitive deficits and symptoms experienced by patients with OCD.

## Introduction

Obsessive compulsive disorder (OCD) is a psychiatric illness prevalent in 1%–2% of adults and is described by the National Institute of Mental Health as typically chronic with a gradual onset (1, 2). OCD is characterized by two major symptoms: obsessions and compulsions (1, 2). The former are defined as disturbing and intrusive thoughts, urges, or impulses, and the latter as recurring behaviors or mental acts that patients feel driven to perform (1). Patients with OCD exhibit a diverse array of obsessions and compulsions that range in severity. The symptoms tend to follow a general order, however. Obsessive thoughts arise producing anxiety. In response to obsessions, compulsions are performed providing temporary anxiety reduction until the intrusive, obsessive thoughts occur again, beginning the cycle anew (1, 2). Obsessions and compulsions are usually thematically linked. For example, patients might fear contamination by dirt or germs, with accompanying trepidation that this could result in serious illness or death to self or others, out of proportion with actual risk (3, 4). The anxiety drives patients to wash or clean excessively, repetitively, or in a ritualistic fashion, which for a time reduces distress. Compulsive washing usually only ends once a *feeling* of cleanliness is achieved through completion of the ritual or after exaggerated washing, rather than following appropriate cleansing and elimination of observable dirt. The feeling of cleanliness is generally fleeting, however, and despite efforts to avoid contamination, actual or perceived exposure to contaminants inevitably recurs and the cycle repeats. Patients with OCD spend substantial amounts of time preoccupied with obsessions and performing compulsions, interfering with employment, goals, and relationships (5). The neural bases of obsessions and compulsions are not fully elucidated.

The striatum, the input region of the basal ganglia, is now extensively implicated in motor and cognitive functions (6, 7). Striatal abnormalities are noted in movement disorders and increasingly in psychiatric illnesses (8–10). The striatum can be divided into at least two sub-regions, the dorsal (DS) and ventral striatum (VS), based on independent dopaminergic and glutamatergic inputs, vascular supplies, and functions (11–13). DS encompasses the majority of the caudate nucleus and putamen. The DS has been implicated in decision-making (14), cognitive flexibility (15), and inhibition of habitual responses (16). In contrast, the VS is comprised of the nucleus accumbens and ventral regions of the caudate nucleus and putamen (10). The VS has been shown to underlie motivation and reward processing, as well as learning associations among stimuli, responses, and rewards (14, 17–20).

Recently, OCD has been linked to deficits in the striatum using evidence from structural and functional magnetic resonance imaging (MRI). Structural MRI studies utilizing voxel-based morphometry have consistently found volumetric differences within the striatum with the consensus being reduced volume of DS (21, 22) and increased volume of VS (21, 23–27). Additionally, volumetric differences have been found in regions reciprocally connected to the striatum, such as the hippocampus, palladium, and thalamus (28, 29). The volumetric abnormalities in OCD are also reflected in resting state basal activity and activity related to cognitive tasks. Positron Emission Tomography (PET) and resting fMRI have found increased glucose metabolism and increased activity in regions of VS compared to controls (30–36). Interestingly, it appears that reversal learning (37) and reward learning (38), cognitive functions mediated by VS, are diminished in OCD patients, coupled with decreased VS activity compared to healthy controls. Conversely, resting state and DS-mediated task activity in DS is reduced compared to controls (32, 39, 40). The task-related DS hypoactivation extends to behavior, as well. Patients with OCD have shown diminished cognitive flexibility and decision-making as evidenced by increased reaction times (RTs) compared to healthy controls (41, 42). The increase in RT may be the result of failure to use the learned information effectively and to refine the responses across subsequent repetitions of the stimuli. Taking everything together, OCD patients seem to have increased volume and baseline activity but decreased task-related activity in VS, and diminished volume and activity in DS.

We have shown that separating trials in a stimulus-response learning paradigm into decision versus learning phases differently engages DS and VS (14, 19, 43). In stimulus-response learning experiments, in each trial a) a stimulus is presented, a response is selected and performed, and b) feedback regarding the accuracy of the response is provided. Response selection and enactment reflect decision-making operations, which engage DS (14, 44, 45). As has been shown previously, decision-making performance improves as participants become more familiar with the stimuli and responses and this is evidenced by improved decision accuracy and reduced response time (RT) across trials (14, 46, 47). Feedback processing is the means through which stimulus-response associations are learned, and this phase correlates with VS activation using neuroimaging (17, 37, 48–50). Learning is examined with changes in accuracy across the learning blocks with more errors early on and diminishing as participants use the feedback provided to update their responses.

Using this stimulus-response association task coupled with fMRI, we specifically investigated DS and VS-mediated cognitive functions in OCD. Patients with OCD evidence difficulty inhibiting habitual responses as well as aberrant stimulus-response association learning (8, 32). We predicted that both DS and VS functions would be impaired in OCD. Behaviorally, we anticipated diminished decision-making performance evidenced by an impaired or diminished reduction in RT across blocks, coupled with reduced DS activity compared to healthy controls. Similarly, we expected a slower rate of change in performance accuracy across blocks and reduced VS activity compared to controls.

## Materials and Methods

### Participants

Fourteen patients with OCD and 16 healthy control participants completed the experiment. All patients with OCD were previously diagnosed by a licensed psychiatrist. Past and current medical histories of patients were reviewed during a telephone screening as well as in person, immediately prior to performing the experimental task. All patients in this study were diagnosed with OCD and followed for this condition by a psychiatrist. Consent was obtained to contact the treating psychiatrist to confirm the diagnosis of OCD and the absence of other psychiatric or known neurological conditions. All participants had no confounding, diagnosed neurological, or psychiatric disorders. Patients abusing alcohol, prescription or street drugs, or taking cognitive-enhancing medications like donepezil, galantamine, rivastigmine, memantine, or methylphenidate were excluded from participating.

Mean group demographics and clinical information for all patients and controls were recorded (**Table 1**). The Yale-Brown Obsessive-Compulsive Scale (YBOCS) was administered to patients with OCD to quantify the presence and severity of obsessive and compulsive symptoms. The YBOCS yields a total OCD severity score, as well as obsession and compulsion sub-scores (**Table 1**).

**Table 1:** Demographic and clinical characteristics for participants in the OCD and control groups.

	OCD	Control	*p* value
Number of participants	14	16	–
Age	26.07 (1.65)	24.50 (0.68)	0.39
Education level	16.92 (0.65)	17.54 (0.45)	0.48
YBOCS–Total Score	18.00 (1.59)	–	–
YBOCS–Obsession sub-score	9.71 (0.85)	–	–
YBOCS–Compulsion sub-score	8.29 (1.08)	–	–
BDI-II	11.64 (2.54)	4.00 (0.95)	0.01*
BAI	9.14 (1.44)	3.00 (0.89)	0.002*
SAS	9.86 (1.25)	8.91 (0.96)	0.58
ANART	121.67 (1.85)	120.88 (1.45)	0.76
Epworth Sleepiness Scale	8.21 (1.30)	5.54 (0.67)	0.10
Oxford Happiness score	3.79 (0.17)	5.08 (0.14)	0.00002*
BIS-11	58.36 (2.64)	56.54 (3.75)	0.73
MoCA	27.86 (0.49)	28.82 (0.40)	0.17

Values are presented as group means and standard error of the mean (SEM) in braces. ANART, National Adult Reading Test IQ Estimation; MOCA, Montreal Cognitive Assessment total score out of 30; BDI-II, Beck Depression Inventory II; BAI, Beck Anxiety Inventory; BIS-11, Barratt Impulsiveness Scale; SAS, Starkstein Apathy Scale; YBOCS, Yale-Brown Obsessive Compulsive Scale. *indicates statistical significance (*p* < 0.05).

All participants provided informed written consent to the protocol before beginning the experiment according to the Declaration of Helsinki. This study was approved by the Health Sciences Research Ethics Board (REB #18517) of the University of Western Ontario.

### Experimental Design

**Figure 1:**
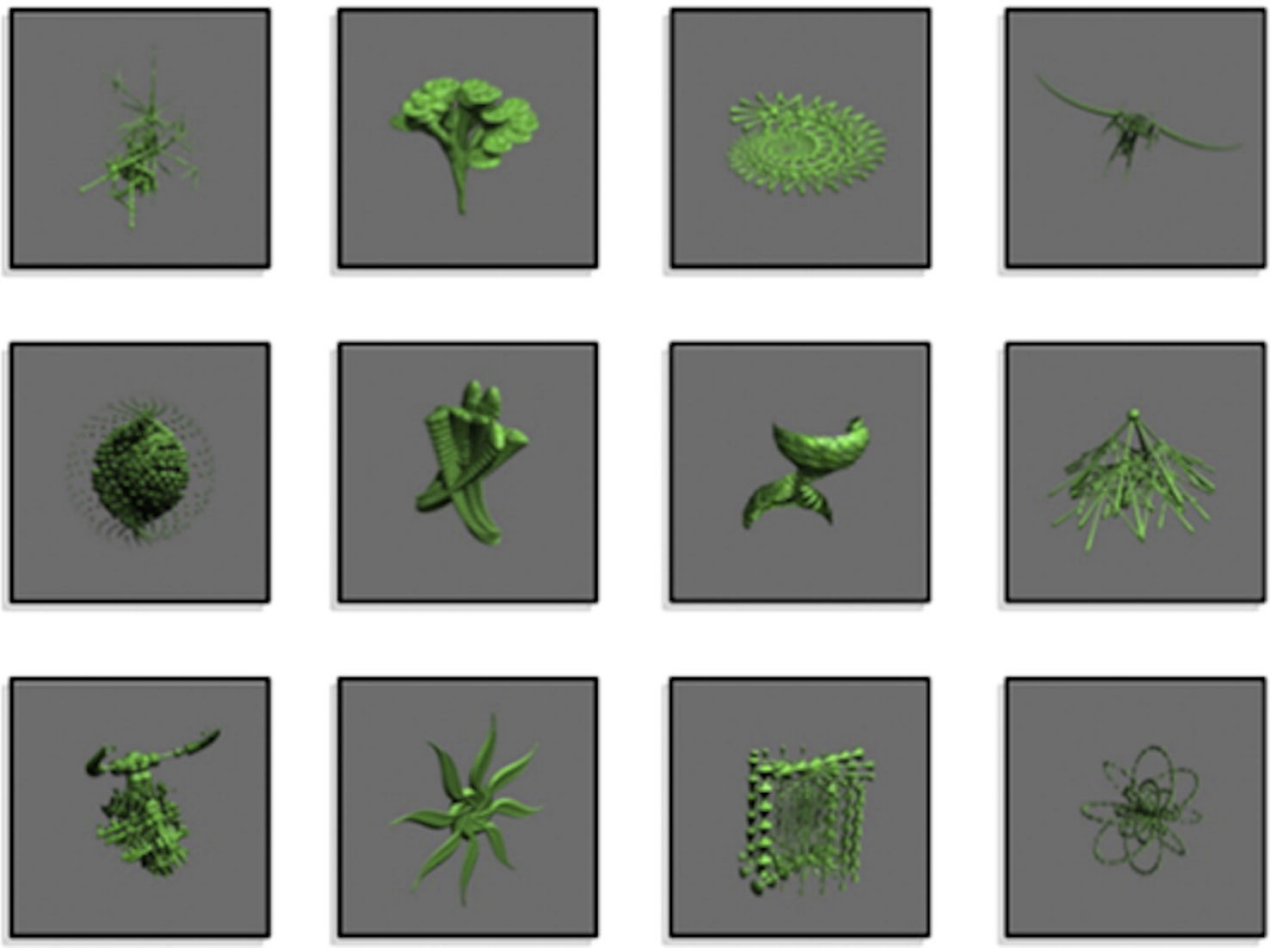
Abstract images presented in the experiment. Images were associated with a button pressed by the index, middle, or ring finger buttons. Modified from NeuroImage, 185, Nole M. Hiebert, Adrian M. Owen, Hooman Ganjavi, Daniel Mendonça, Mary E. Jenkins, Ken N. Seergobin, Penny A. MacDonald, Dorsal striatum does not mediate feedback-based, stimulus-response learning: An event-related fMRI study in patients with Parkinson's disease tested on and off dopaminergic therapy, 455–470, Copyright (2019), with permission from Elsevier.

Each participant completed a stimulus-response task in which they learned to associate 12 abstract images with one of three button-press responses. These images, shown in **Figure 1**, were computer-generated with GroBoto (Braid Art Labs, Colorado Springs, USA). The task was administered within a 3 Tesla (T) fMRI scanner to observe concurrent regional activity within the striatum.

**Figure 2:**
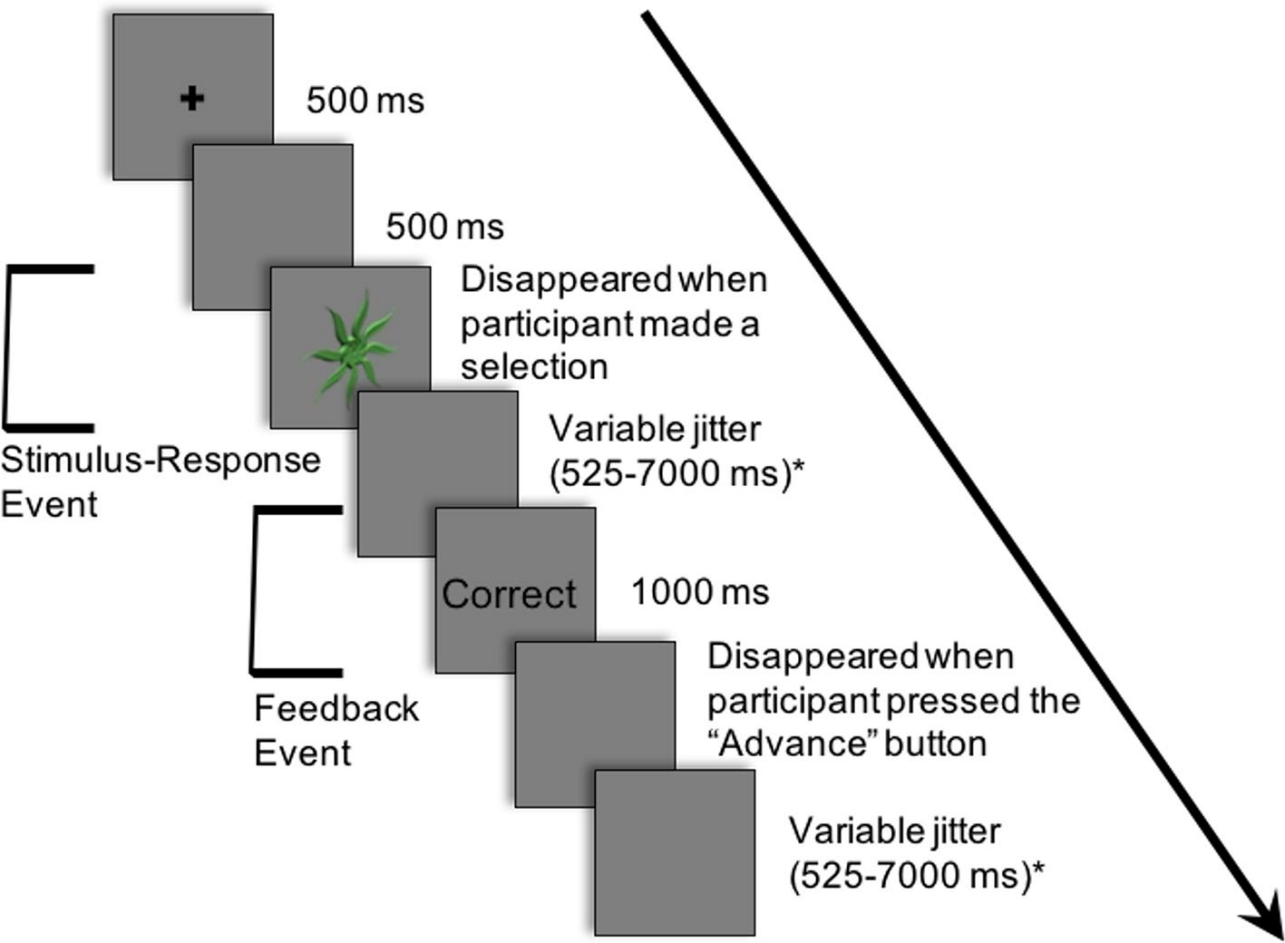
Example of a single trial in the experiment. Participants learned to associate 12 abstract images with one of three button-press responses. The following is an example of a trial: (i) a cross appeared in the center of the projection screen for 500 ms; (ii) a blank screen occurred for 500 ms; (iii) an abstract image was presented in the center of the projection screen until a button-press response; (iv) a blank screen appeared for a variable period of time sampled from an exponential distribution (mean: 2,500 ms; minimum: 525 ms; maximum: 7,000 ms); (v) feedback (i.e., “Correct” or “Incorrect”) appeared for 1,000 ms; (vi) a blank screen appeared for a variable period of time sampled from an exponential distribution (mean: 2,500 ms; minimum: 525 ms; maximum: 7,000 ms). Modified from NeuroImage, 185, Nole M. Hiebert, Adrian M. Owen, Hooman Ganjavi, Daniel Mendonça, Mary E. Jenkins, Ken N. Seergobin, Penny A. MacDonald, Dorsal striatum does not mediate feedback-based, stimulus-response learning: An event-related fMRI study in patients with Parkinson's disease tested on and off dopaminergic therapy, 455–470, Copyright (2019), with permission from Elsevier. *The inter-stimulus and inter-trial intervals (ISI and ITI, respectively) were jittered between the response and feedback and between the offset of feedback and the beginning of the subsequent trial to create two fMRI events within each trial: a) the Stimulus-Response Decision Event and b) the Feedback Event.

**Figure 2** demonstrates an example of an experimental trial. Each trial consisted of an abstract image being presented in the centre of a projection screen until a response was selected. The participant chose one of the three button-press options. Deterministic feedback regarding accuracy of the response (i.e., “Correct” or “Incorrect”) was presented. This provided the basis for learning the stimulus-response associations between each abstract image and the corresponding button-press response.

Trials were organized into five blocks. Each block was comprised of 24 trials—with each abstract image randomly appearing twice within each block. After each block, a percentage score of the number of correct responses was displayed—indicating performance for the block.

There were four buttons on the button box. The second, third, and fourth buttons each corresponded to two of the twelve abstract images. Participants pressed these three buttons with their index, middle, and ring fingers, respectively. The first button, pressed by the thumb, served to advance from the feedback phase to the next trial. In this way, a motor response was included in both decision-making and feedback phases of each trial.

Trials proceeded as follows: (i) a cross appeared in the center of the projection screen for 500 ms; (ii) a blank screen occurred for 500 ms; (iii) an abstract image was presented until a button-press response was made; (iv) a blank screen appeared for a variable period of time (mean: 2,500 ms; minimum: 525 ms; maximum: 7,000 ms); (v) feedback (i.e., “Correct” or “Incorrect”) appeared for 1,000 ms; (vi) a blank screen appeared until the participant pressed the first button with his/her thumb to proceed to the next trial; (vii) a blank screen appeared for a variable period of time (mean: 2,500 ms; minimum: 525 ms; maximum: 7,000 ms).

The inter-stimulus interval (ISI), the period between the response selection and feedback, and the inter-trial interval (ITI), the duration between the offset of feedback and the onset of the following trial, were jittered. These intervals varied in duration, and the length of both the ISI and ITI was sampled from an exponential distribution (mean: 2,500 ms; minimum: 525 ms; maximum: 7,000 ms) on each trial.

The variable rest intervals served to distinguish two independent events within each trial: a) the Stimulus-Response Decision Event and b) the Feedback Event (**Figure 2**). As previously discussed, in the Stimulus-Response Decision Event, an abstract image is presented until a button-press response is performed. The Feedback or Learning Event consisted of the period during which feedback was provided up until the participant indicated their willingness to advance to the next trial with a button-press response.

## Behavioral Data Analysis

### Measure of Decision-Making

In each of the five blocks, each stimulus was presented twice. During the first few blocks of the session (i.e., Blocks 1–3), participants are actively learning the stimulus-response associations. Responses are quite error-prone as participants are acquiring these relations through trial and error, and response times (RTs) are highly variable. Towards the end of the Session, when much of the learning has already taken place (i.e., Blocks 4–5), the time to respond with the correct button-press decreases. RTs during the final block reflect deliberation, and therefore measure the efficiency of decision-making processes (14). Comparing RTs for accurately-performed responses to the first presentation of each stimulus in the final block of the Session (i.e., Block 5), with RTs for accurately-performed responses to the second presentation of each stimulus in the penultimate block of the Session (i.e., Block 4), provided our measure of stimulus-response decision-making. No new feedback-based learning occurs from correctly-performed responses for second presentation of stimuli in Block 4 to correctly-performed responses for the first presentation of these stimuli in Block 5, emphasizing decision-making processes. Further, weighting by RT in Block 4 accounted for individual differences in participant RTs. Consequently, Final Block RT Change Scores, calculated as RT for accurate responses to first presentation of stimuli in Block 5 minus RT for accurate responses to second presentation of stimuli in Block 4, was our measure of decision-making efficiency. Independent *t*-tests were conducted on Final Block RT Change Scores between OCD patients and healthy controls.

### Measure of Stimulus-Response Association Learning

The rate of change, or slope, of response accuracy (%) recorded after each block across the five blocks of the session was used to operationalize the rate at which participants learned the stimulus-response associations. Block 0 was included in the calculation with a value of zero, as participants are assumed to have no prior learned association between the abstract images and the correct button-press responses at the outset. The equation used to calculate Learning Slope was the standard slope of the linear regression function (Microsoft Excel, 2011):

$$b=\frac{\sum \left(x-\bar{x}\right)\left(y-\bar{y}\right)}{\sum {\left(x-\bar{x}\right)}^{2}}$$

where *b* is the slope, and *x* and *y* are the sample means of the number of blocks and block scores, respectively. Statistical analysis involved conducting an independent, unpaired *t*-test on Slope of Learning scores between OCD patients and healthy controls.

### Imaging Acquisition

FMRI data were collected in a 3T Siemens Magnetom Prisma with Total Imaging Matrix MRI at Robarts Research Institute at the University of Western Ontario. A scout image was taken to properly orient the participant and T_1_ for anatomical localization. Five runs of T_2_*-weighted functional acquisitions were completed, each consisting of one block with 24 trials. Each run lasted approximately 5 min. A whole brain image was taken every 2.5 s, each consisting of 43, 2.5 mm-thick slices. The field of view was oriented along the anterior and posterior commissure of the brain with a matrix of 88 × 88 pixels. Each isotropic voxel size was 2.5 × 2.5 × 2.5 mm^3^. The echo time was 30 ms and the flip angle was 90˚.

## FMRI Data Analysis

Statistical Parametric Mapping version 12 (SPM12; Wellcome Department of Imaging Neuroscience, London, United Kingdom) was used in conjunction with Matrix Laboratory (MATLAB, Mathworks, Inc., Natick, Massachusetts, United States) to complete fMRI analysis. The scans were slice-time corrected, reoriented for participant motion, spatially normalized to the standard Montreal Neurological Institute (MNI) template, smoothed with an 8-mm full-width, half maximum Gaussian kernel, and high-pass filtered (0.0056 Hz).

Fixed-effect analyses in SPM12 were used to model each participant's data. Regressors were generated by convolving onsets and durations of Stimulus-Response Decision, Feedback, and ITI Rest Events with the canonical hemodynamic response function. The Stimulus-Response Decision Event was demarcated as the time between onset of abstract image presentation and button-press response. The Feedback Event comprised the time of feedback presentation, lasting 1,000 ms and then until the participant pressed the thumb button to proceed to the next trial. As a result, motor responses occurred in both Stimulus-Response Decision and Feedback Events. To reiterate, the ITI Rest Event includes the variable period of time sampled from an exponential distribution (mean: 2,500 ms; minimum: 525 ms; maximum: 7,000 ms) after the post-feedback button-press until the beginning of the next trial. While it is termed rest, it is important to note that is it not a *true* rest period in that the participant may be completing other cognitive processes such as processing the previous reward, rehearsing stimulus-response associations, and anticipating future rewards. Nevertheless, the use of this period as a baseline with which to compare task-relevant events has been used previously (19, 43, 50). A general linear model (GLM) was created and included the regressors for the Stimulus-Response Decision, Feedback, and ITI Rest Events. The GLM examined regional blood-oxygenation-level dependent (BOLD) activity associated with these events.

The Harvard-Oxford Subcortical Atlas in the FMRIB Software Library version 5.0 (FSL v5.0; Analysis Group, FMRIB, Oxford, United Kingdom) was used to define striatal regions. MNI space was used as an *x*, *y*, and *z* coordinate system to delineate each region. The VS was defined as *z* < 2 in MNI space, including the nucleus accumbens and the ventral portion of the caudate nucleus and putamen (51). The DS was defined as *z* ≥ 2 in MNI space, consisting of the bulk of the caudate nucleus and putamen (51).

### Replication Contrasts

First, group-level contrasts examined brain activity correlating with Stimulus-Response Decision and Feedback Events, collapsed across Group (OCD and control) to confirm that we replicated the results from our previous studies (14, 19, 43) as follows:

- Stimulus-Response Decision versus Feedback Events collapsed across Group (OCD and control)

Peaks in these contrasts had an extent threshold of at least 10 contiguous voxels and are reported at a significance level of p < 0.05 corrected for multiple comparisons using familywise error correction (FWE) at the voxel level.

## OCD Versus Control Contrasts

Subsequently, events of interest were contrasted between Group (OCD versus control). The contrasts of interest were:

- OCD versus control for Stimulus-Response Decision Events
- OCD versus control for Feedback Events

For these contrasts between OCD patients and controls, peaks had an extent threshold of at least 10 contiguous voxels and are reported at a significance level of p < 0.05 FWE corrected for multiple comparisons at the voxel level.

### Correlation Analysis

Next, we investigated brain-behavior correlations to confirm that behavioral performance was related to DS and/or VS activity patterns. We tested whether BOLD signal in striatal regions correlated with behavioral indices of response selection decisions and learning respectively. Specifically, we tested whether activity in DS and VS ROIs were correlated with our measure of decision-making, Final Block RT Change, and our measure of learning efficiency, Learning Slope. Correlations were performed separately for OCD and healthy control groups in the event that response selection performance and learning performance differed across groups. A right and left DS ROI and a right and left VS ROI were created, encompassing the entire DS and VS, respectively, using the MarsBar Toolbox in SPM12 (52). Beta values in our ROIs were extracted from two contrasts of interest described below.

- Stimulus-Response Decision Events minus Rest
- Feedback Events minus Rest

The beta values for each ROI were correlated with behavioral measures of stimulus-specific response selection (i.e., the Final Block RT Change) and learning (i.e., Learning Slope) relative to rest for each group separately.

Additionally, Bayesian analysis was performed on the brain-behavior correlations to investigate the strength of negative results. Briefly, the application of Bayesian analysis reduces pitfalls in dealing with negative results and interpreting null effects. Bayesian analysis treats null and alternative hypotheses symmetrically, using the data themselves to determine the relative fit to the respective models. In this way, the statistical obstacles and validity of accepting versus rejecting the null hypotheses are equated with Bayesian analysis (53). If the Bayes' factor of the average beta values is <3, it strongly supports the null hypothesis, that there is no significant correlation (53).

## Results

### Demographic and Clinical Characteristics of OCD Patients and Healthy Controls

Demographic and clinical characteristics of each group are presented in **Table 1**. The mean [standard error about the mean (SEM)] ages of the patient and control groups were 26.07 (1.65) and 24.50 (0.68), respectively. The mean years of education (SEM) of the patient and control groups were 16.93 (0.66) and 17.55 (0.45), respectively. There were no significant differences between OCD and control participants (see **Table 1**) in demographic or cognitive data. Participants with OCD scored significantly higher on Beck Depression Inventory II, Beck Anxiety Inventory, and significantly lower Oxford Happiness Questionnaire compared to controls, as would be expected given the nature of OCD. Out of the 14 patients with OCD, 6 patients were on a stable dose of a selective-serotonin reuptake inhibitor (SSRI), one patient was on a stable dose of a benzodiazepine, and 7 were on no medication for their OCD. YBOCS was administered to OCD patients only. Again, the YBOCS measures the presence and severity of obsessive and compulsive symptoms. The scale yields a total score as well as a sub-score for obsessions and compulsions, although only the total score is interpreted clinically. The current OCD cohort had a mean total score of 18 which suggests moderately severe OCD (54). YBOCS total scores ranged from 8 (mild OCD) to 26 (severe OCD), suggesting a wide range OCD severity in our study (54–56).

### Behavioral Data

Behavioral measures of decision-making and feedback-based learning are presented in **Table 2**.

**Table 2:** Behavioral measures for patients with OCD and control participants.

	Final Block Accuracy (%)	Final Block Mean RT (ms)	Final Block RT Change (ms)	Slope of Learning
OCD	76.20 (5.74)	1316.72 (138.10)	153.74 (91.87)	0.085 (0.015)
Control	84.72 (3.72)	1251.05 (97.84)	−190.06 (123.16)	0.132 (0.016)

Values are presented as group means and SEM in braces. Final Block RT Change is a difference score between the mean RT of the first presentation of each of the stimuli that were associated with correct responses of Block 5 and the mean RT of the second presentation of each of the stimuli that were associated with correct responses of Block 4. Slope of Learning was calculated using the slope of the linear regression function in Microsoft Excel (2011).

### Measure of Decision-Making

**Figure 3:**
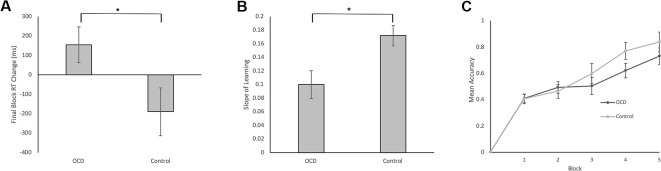
Behavioral Data in Patients with OCD and Healthy Controls. **(A)** Final Block RT Change was our measure of decision-making efficiency. It was calculated by subtracting the mean RT for correct events of the first presentation of the stimuli in Block 5 from the mean RT for correct events of the second presentation of the stimuli in Block 4. We found significantly less reduction in Block 5 RT relative to Block 4 RT for OCD patients compared to controls (t = 1.90, p = 0.033). **(B)** Slope of Learning served as a measurement of learning efficiency. To reiterate, Slope of Learning was calculated using the block accuracy scores over five blocks using the slope of the linear regression function (Microsoft Excel, 2011). Slope of Learning was significantly slower in OCD patients compared to healthy controls (t = 2.53, p = 0.008). **(C)** Mean accuracy plotted across blocks for patients with OCD and healthy controls. No significant differences arose between groups across blocks 1–5. Error bars represent SEM. *p < 0.05.

Final Block RT Change Scores, calculated as RT for accurate responses to the first presentation of each stimulus in Block 5 minus RT for accurate responses to the second presentation of each stimulus in Block 4, was our measure of decision-making efficiency. By end of Block 4 and throughout Block 5, stimulus-response associations are expected to be well learned. In fact, no new feedback-based learning occurs from correctly performed responses for second presentation of stimuli in Block 4 to correctly performed responses for the first presentation of these stimuli in Block 5. These Final Block RT Change Scores, therefore, emphasize decision-making processes isolated from learning processes. Further, weighting by RT in Block 4 accounts for individual differences in participant RTs. We found significantly less improvement from Block 4 to Block 5 in terms of the Final Block RT Change Score for OCD patients compared to controls (*t* = 1.90, *p* = 0.033; **Figure 3A**). In fact, the score was positive for OCD patients, meaning that they slowed down in Block 5 relative to Block 4, whereas controls demonstrated the expected reduction of RT, reflecting more efficient decision-making, requiring less deliberation by Block 5. It is important to note, however, that mean RT and accuracy in the final block did not differ significantly between OCD patients and controls (Final Block Mean RT: *t* = 0.53, *p* = 0.701; Final Block Mean Accuracy: *t* = 0.76, *p* = 0.226).

### Measure of Stimulus-Response Association Learning

Efficiency of stimulus-response association learning was estimated using the Slope of Learning defined as the rate of change of response accuracy over five blocks of stimulus-response trials. Slope was calculated using the slope of the linear regression function in Microsoft Excel (2011). An independent sample t-test on Slope of Learning scores was conducted between OCD and control participants. We found significantly slower learning, with shallower slope, in patients with OCD compared to control participants (*t* = 2.53, *p* = 0.008; **Figure 3B**). When accuracy scores were broken down by block and between group differences were examined, no significant differences arose across any individual block (**Figure 3C**).

### FMRI Data

**Figure 4:**
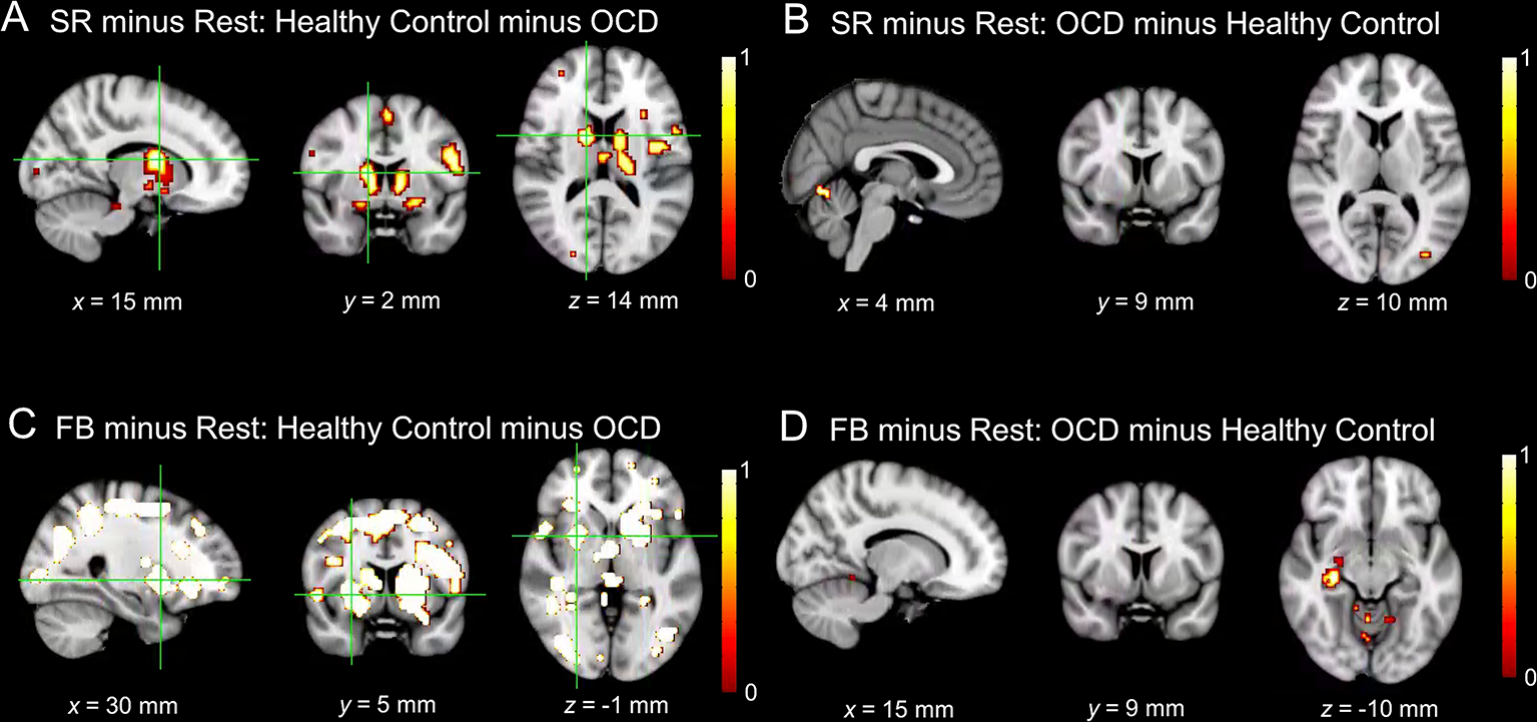
Significant activations in contrasts of interest comparing healthy controls and patients with OCD. Activation maps are presented at a threshold of p < 0.001 uncorrected for multiple comparisons to allow for visualization of activation in all contrasts. Areas in grey and red (0) reflect regions of the brain with little activation and areas in yellow reflect maximal activation (1). **(A)** BOLD signal for healthy control minus OCD patients for Stimulus-Response Decision Events minus Rest. Significant activity occurred in the bilateral dorsal caudate nuclei (peak coordinates: 15, 2, 14; t = 5.32, p = 0.001 FWE, and peak coordinates: −12, −1, 8; t = 4.64, p = 0.019 FWE). **(B)** BOLD signal for OCD patients minus healthy controls for Stimulus-Response Decision Events minus Rest. No significant activity arose in the striatum. **(C)** BOLD signal for healthy controls minus OCD patients for Feedback Events minus Rest. Significant activity arose in bilateral ventral putamina (peak coordinates: 30, 5, −1; t = 5.61, p < 0.001 FWE, and peak coordinates: −27, 2, −1; t = 5.05, p = 0.004 FWE), as well as left dorsal putamen (peak coordinates: −27, −1, 11; t = 5.67, p < 0.001 FWE). **(D)** BOLD signal for OCD patients minus healthy controls for Feedback Events minus Rest. No significant activity arose in the striatum. N.B. SR, Stimulus-Response Decision Events; FB, Feedback Events in the figure.

**Figure 5:**
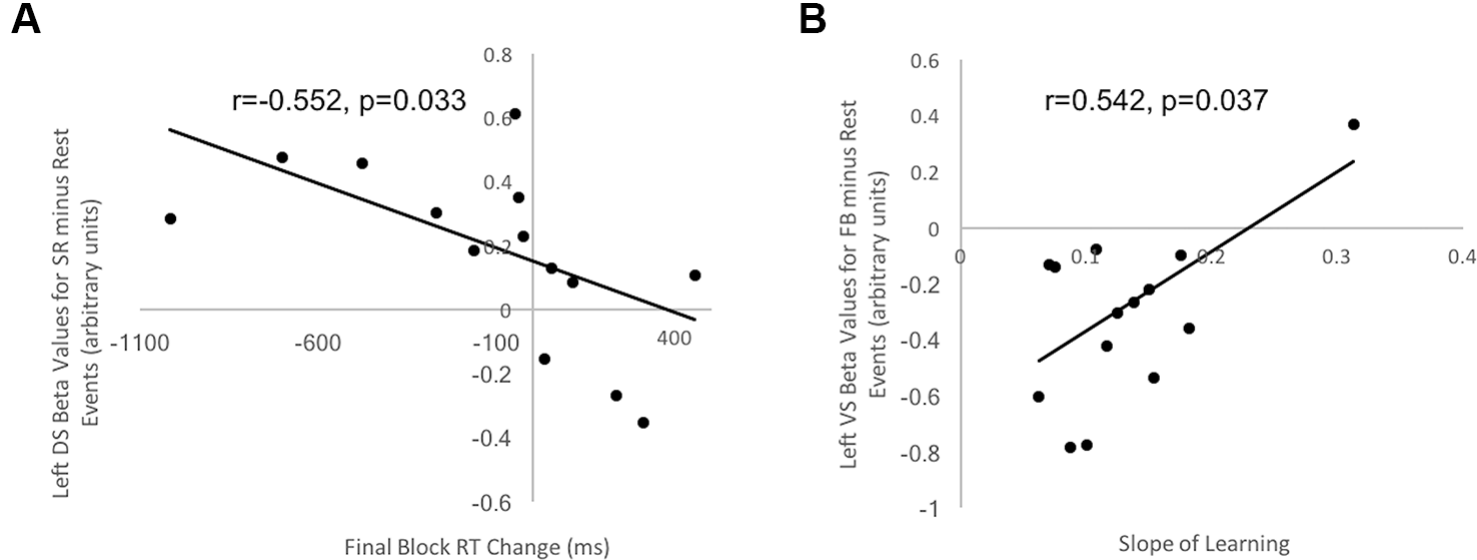
Correlation between behavioral indices of decision-making and learning for control participants and beta values in striatal ROIs. **(A)** Correlation between Final Block RT Change and beta values in left DS ROI for Stimulus-Response Decision Events minus Rest in healthy controls was negative and significant (r = −0.552, t = 1.99, p = 0.033). **(B)** Correlation between Slope of Learning and beta values in left VS ROI for Feedback Events minus Rest in healthy controls was positive and significant (r = 0.542, t = 1.93, p = 0.037). No other correlations for healthy controls and no correlations between beta values extracted from striatal ROIs and behavioral measures for OCD patients were significant.

Significant activations in contrasts of interest are presented in **Tables 3**, **4**, and **Figures 4**, **5**. Contrasts are reported at a significance level of *p* < 0.05 FWE, unless otherwise indicated.

**Table 3:** Significant brain activations in contrasts of interest collapsed across Group (OCD and control) reported in MNI space.

Contrast	Anatomical Area	Cluster Size	*t*	*p*FWE	*x, y, z*
SR minus FB	**Left Dorsal Putamen**	442	4.90	0.004	−24, −1, 8
	Right Angular Gyrus	342	5.70	<0.001	54, −43, 23
	Right Central Opercular Cortex	157	4.90	0.019	42, −1, 17
	Left Cerebral White Matter	14	4.37	0.035	−27, −22, 32
FB minus SR	**Right Ventral Caudate**	10	4.15	0.079	9, 5, −2
	Left Thalamus	192	7.53	<0.001	−21, −22, 5
	Left Insula	94	6.22	<0.001	−33, 23, 2
	Right Insula	71	5.71	<0.001	33, 23, −4

Cluster size is reported in voxels. Coordinates are reported in MNI space. Striatal regions are presented first and highlighted in each contrast. N.B. SR, Stimulus-Response Decision Events; FB, Feedback Events.

**Table 4:** Significant brain activations in patients with OCD versus healthy controls in contrasts of interest reported in MNI space.

Contrast	Anatomical Area	Cluster Size	*t*	*p*FWE	*x, y, z*
SR Events					
Control minus OCD	**Right Dorsal Caudate**	21	5.32	0.001	15, 2, 14
	Left Insular Cortex	48	6.33	<0.001	−30, 26, 5
	Right Insular Cortex	19	5.26	0.001	33, 26, 2
OCD minus control	No suprathreshold activations				
FB Events					
Control minus OCD	**Right Ventral Putamen**	28	5.61	<0.001	30, 5, −1
	**Left Ventral Putamen**	**	5.05	0.004	−27, 2, −1
	**Left Dorsal Putamen**	113	5.67	<0.001	−27, −1, 11
	Left Lateral Occipital Cortex	56	5.81	<0.001	−42, −70, −4
OCD minus control	No suprathreshold activations				

Cluster size is reported in voxels. Coordinates are reported in MNI space. Striatal regions are presented first and highlighted in each contrast. **Cluster size unobtainable as peak coordinates are within a larger cluster. N.B. SR, Stimulus-Response Decision Events; FB, Feedback Events.

### Replication Contrasts

Significant activations in these contrasts of interest are found in **Table 3**.

#### Stimulus-Response Decision versus Feedback Events

Significant activity arose in the DS, specifically in the left dorsal putamen in the Stimulus-Response Decision minus Feedback Events contrast (peak coordinates: −24, −1, 8; *t* = 4.90, *p* = 0.004 FWE). In the Feedback minus Stimulus-Response Decision Events contrast, activity trended towards being significant in the right ventral caudate of the VS (peak coordinates: 9, 5, −2; *t* = 4.15, *p* = 0.079 FWE).

### OCD Versus Control Contrasts

Significant activations in these contrasts of interest are found in **Table 4**.

#### Stimulus-Response Decision Events: Control Minus OCD

There was greater activity in the right dorsal caudate nucleus of the DS (peak coordinates: 15, 2, 14; *t* = 5.32, *p* = 0.001 FWE; **Figure 4A**) in healthy controls compared to patients with OCD when examining Stimulus-Response Decision Events.

#### Stimulus-Response Decision Events: OCD Minus Control

No activity occurred in the striatum at *p* < 0.05 FWE, or even at the liberal threshold of *p* < 0.001 uncorrected when OCD Stimulus-Response Decision Events were contrasted with control events (**Figure 4B**).

#### Feedback Events: Control Minus OCD

There was greater activity in bilateral ventral putamina of the VS (peak coordinates: 30, 5, −1; *t* = 5.61, *p* < 0.001 FWE, and peak coordinates: −27, 2, −1; *t* = 5.05, *p* = 0.004 FWE), as well as left dorsal putamen (peak coordinates: −27, −1, 11; *t* = 5.67, *p* < 0.001 FWE) in healthy controls compared to patients with OCD when examining Feedback Events (**Figure 4C**).

#### Feedback Events: OCD Minus Control

No activity occurred in the striatum at *p* < 0.05 FWE, or even at the liberal threshold of *p* < 0.001 uncorrected in the OCD minus control Feedback Events contrast (**Figure 4D**).

### Brain-Behavior Correlations: OCD and Controls Separately

One right and one left ROI encompassing the entirety of the DS, and one right and one left ROI encompassing the entirety of the VS were created for the brain-behavior correlations. Beta values for each of the four ROIs were extracted from Stimulus-Response Decision Events minus Rest and Feedback Events minus Rest across all five blocks. These were correlated with Final Block RT Change and Slope of Learning in controls and OCD patients separately.

#### Striatum and Decision-Making Efficiency

Final Block RT Change scores, our measure of decision-making efficiency, were correlated with beta values from each of the two DS and VS ROIs, separately for healthy controls and OCD patients. For control participants, a significant and negative correlation occurred between Final Block RT Change Score and beta values in the left DS ROI *during Stimulus-response Decision Events minus Rest* (*r* = −0.552, *t* = 1.99, *p* = 0.033, uncorrected for multiple comparisons; **Figure 5A**). This suggested that those participants with greater activity in the left DS had greater reduction of their response selections and enactments, independent of new learning, than those with lesser DS activation. No significant correlation arose in control participants for our decision-making efficiency score and BOLD signal in either DS or VS *during Feedback Events minus Rest*. For OCD patients, our decision-making efficiency score did not correlate with neural activity in DS or VS, during Stimulus-Response Decision or Feedback Events minus Rest. Additionally, all Bayes factors involving OCD beta values were <1, which strongly supports the null hypothesis that no correlation exists.

#### Striatum and Learning From Feedback

Learning Slope was correlated with beta values from each of the VS and DS ROIs, separately for controls and OCD patients. Looking at the control data, a significant, positive correlation occurred between Slope of learning and beta values extracted from the left VS ROI (*r* = 0.542, *t* = 1.93, *p* = 0.037, uncorrected for multiple comparisons; **Figure 5B**) for the Feedback Event minus Rest contrast. No significant or trending correlations were present in the control participants' data relating Learning Slope and BOLD signal during Stimulus-response Decision Events minus Rest. For OCD patients, Learning Slope, our measure of learning efficiency, did not correlate with neural activity during either Feedback or Stimulus-Response Decision Events minus Rest. Additionally, all Bayes factors involving OCD beta values were <1, which strongly supports the null hypothesis that no correlation exists.

## Discussion

### Overview of Findings

Cognitive functions mediated by DS and VS were investigated in OCD. Replicating our previous studies, we found that DS BOLD signal correlated with stimulus-specific response selection and enactment, and not with learning from feedback (14, 19, 43). Patients with OCD evidenced less efficient decision-making relative to healthy controls, even when controlling for new feedback-based learning and baseline individual differences in RT, and though both groups ultimately achieved similar levels of accuracy at the end of five blocks of stimulus-response learning. Though healthy age-matched controls made decisions more quickly in Block 5 relative to Block 4, OCD patients did not show this reduction in decision-making RT. Correspondingly, OCD patients had lower DS activation during stimulus-response decisions relative to controls. Finally, though control participants with higher DS activation during Stimulus-Response Decision Events evidenced greater decision efficiency (i.e., higher Final Block RT Change Scores), there was no correlation between level of DS activation and decision efficiency in OCD patients.

We also replicated our observation that VS BOLD signal correlated with stimulus-response learning from feedback (14, 19, 43). Patients with OCD learned stimulus-response associations less efficiently than healthy controls, demonstrating a shallower Slope of Learning. In keeping with this, patients with OCD had lower VS BOLD signal corresponding with Feedback Events than healthy age-matched controls. Finally, though control participants with higher VS activation during Feedback Events relative to Rest learned stimulus-response associations more efficiently, there was no correlation between VS activation and speed of acquiring stimulus-response associations through feedback in patients with OCD.

In summary, OCD patients made decisions less efficiently and had reduced DS activation, as well as learned stimulus-response associations more slowly and evidenced lower VS BOLD signal than healthy controls. These results suggest that cognitive impairment in OCD could be mediated by striatal dysfunction as discussed further below and related to the broader literature in OCD.

### Striatum in OCD

Functional changes within the striatum are suspected to have a role in cognitive dysfunction and symptoms of OCD (31, 32, 57). We directly investigated these notions with a methodology that distinguishes DS versus VS functions coupled with fMRI in OCD.

DS has been shown convincingly to mediate decision-making, specifically promoting cognitive flexibility, selecting correct responses even when this requires resisting more habitual responding (42, 58–65). In line with these postulated functions of DS and a number of previous studies (31, 32, 42), patients with OCD in our study demonstrated less efficient decision-making and corresponding DS hypoactivity. Patients with OCD have been shown to have diminished DS function in tasks examining cognitive flexibility (32, 66), response inhibition (32, 67), and generally when making decisions in ambiguous contexts, such as in the Iowa Gambling Task [IGT; (68–70)]. In the IGT, the object is to win as much money as possible by selecting cards from various decks. Some decks of cards are associated with high winnings but also high losses and are disadvantageous in the long run (i.e., “bad decks”), whereas other decks yield more modest winnings and losses but are more advantageous (i.e., “good decks”) over time. Participants are not informed of the distribution of winnings in each of the decks and must figure out which decks are more advantageous through trial and error. Heathy controls typically initially sample each of the decks but will quickly discover which decks are “good” and will continue to choose them. Patients with OCD, on the other hand, have been shown, repeatedly, to persevere with the “bad decks” and win less money overall (68, 69, 71–73). According to previous studies, this dysfunctional decision-making is driven by the prospect of immediate, potentially high rewards and patients with OCD are insensitive to the future consequences of high losses (68).

Nakao et al. (42) investigated decision-making in OCD using Stroop facilitation and interference. In the color-word Stroop task, color words (e.g., RED, BLUE, GREEN) are presented in font colors that are either congruent (e.g., the word RED appears in red font) or incongruent (e.g., the word RED appears in green font) with the color word. Word reading is more habitual than color naming, leading to faster color naming in the congruent condition (i.e., Stroop facilitation) but slower color naming in the incongruent condition (i.e., Stroop interference) relative to naming the color of a neutral letter string. Patients with OCD exhibited greater Stroop interference and reduced DS BOLD signal than healthy controls, consistent with inefficiency in selecting accurate responses and inhibiting incorrect responses, especially when the incorrect response is more practiced and habitual. DS dysfunction and corresponding cognitive inflexibility could lead to the inability to choose naturally rewarding behaviors over compulsive actions (66, 74–76), perpetuating the illness.

Additionally, decision-making deficits seen in OCD can be related to pathological doubt, a common phenomenon seen in OCD. Studies have shown that patients with OCD often experience uncertainty when making decisions due to a lack of confidence in their own perception, attention, and memory (77–79). In decision-making tasks, this can manifest as longer response times compared to healthy controls (77).

VS has been shown to mediate association learning, reward processing, as well as motivation (17, 37, 48–50, 80–82). In the current study, stimulus-response learning was impaired in patients with OCD related to *decreased task-relevant VS activity* during Feedback Learning Events. Our results are consistent with other studies that have shown diminished reversal learning (37) and reward learning (38) and reduced VS activation in OCD patients. In our study, *task-relevant VS hypoactivity* during Feedback Events could be related to baseline hyperactivity in VS. This pattern is supported by the larger literature as well, with higher VS metabolism at rest (32, 83), and in response to symptom-provoking stimuli measured with positron emission tomography (84) in OCD patients relative to controls, despite reduced VS activation during VS-mediated functions such as reward anticipation (57), reward processing, and learning (8, 31, 32, 57). Impairments in striatal-mediated learning has been purported as a significant contributor to the neurological foundations of obsessions and compulsions in OCD patients (37, 75). In a number of studies, in fact, obsessive-compulsive symptoms have been linked to hyperactivity in the VS at baseline (30–36, 57).

A proposed model of OCD suggests that obsessions and compulsions arise due to disproportion between activity levels in VS and DS (57, 85). Baseline VS hyperactivity, presumably related to obsessive thinking and hypervigilance regarding obsession-related stimuli, reduces the impact of VS signals related to appropriate and natural rewards, feedback/outcomes, and motives. The latter leads to reduced capacity for learning appropriate associations and responses (30–36, 57). In essence, the reward system, of which the VS is central, is dysfunctional in OCD patients to regard anxiety-reduction following performance of compulsions as abnormally more rewarding than natural rewards and stimuli [i.e., food, sex; (32, 37, 57)]. Concurrently, hypoactivity in DS results in deficits in cognitive flexibility and decision-making, leading to habitual responses in the form of compulsive behaviors (74–76). Together, these striatal changes presumably interfere with learning of adaptive responses through goal-directed behaviors, and cause impairment in switching away from obsessive thoughts and in inhibiting maladaptive compulsive, habitual behaviors (32, 66, 67, 74–76). Our results are entirely supportive of these models of OCD.

The study is limited by its relatively small sample size (n_OCD_ = 14; n_ctrl_ = 16), however between group differences, corrected for multiple comparisons using familywise error correction, were demonstrated increasing confidence in the results. A secondary limitation with respect to the small sample size of OCD patients in particular is the inclusion of patients with differing severities of OCD and on different medication regimes which could reduce observed power. The OCD sample had YBOCS scores ranging from 8 to 26 which are all considered clinical OCD (55, 56), mild and severe, respectively. Including a more homogenous sample with a smaller variability of YBOCS scores and medications could increase the between group differences, however, the aim of the study was to compare OCD in general to healthy controls and it is expected that all patients diagnosed clinically with OCD would exhibit some level of striatal dysfunction. Additionally, medication was not controlled and half of the patients with OCD were on pharmacotherapy to treat their OCD including SSRIs, benzodiazepine or both, and the other half were on no medication to treat their OCD. Given the small numbers of those on and off medication and the varying classes of medication, we were unable to conduct any contrasts between these groups to investigate any effects of medication on these cognitive functions. Lastly, given the size of the sample, it was impossible to compare subtypes within the OCD group (i.e., contamination, hoarding, ordering, checking, etc.) as differences in neuropathophysiology of each subtype have been reported (4). Further research with a larger sample is necessary to examine the role of the striatum on OCD subtype.

## Conclusions

This study provides strong support for cognitive deficits in DS-mediated decision-making as well as in VS-mediated learning that arise in patients with OCD. Future investigations are needed to investigate the striatal basis for cognitive deficits as well as in inducing and sustaining symptoms of OCD.

## Data Availability Statement

The raw data supporting the conclusions of this article will be made available by the authors, without undue reservation, to any qualified researcher.

## Ethics Statement

The studies involving human participants were reviewed and approved by the Health Sciences Research Ethics Board, University of Western Ontario. The patients/participants provided their written informed consent to participate in this study.

## Author Contributions

NH, KS, and PM contributed conception and design of the study. HG and MW evaluated and referred patients for the study. ML and NH performed the statistical analysis. NH and ML wrote the first draft of the manuscript. HG, MW, AO, and PM wrote sections of the manuscript. All authors contributed to manuscript revision, read, and approved the submitted version.

## Funding

This work was supported by a Natural Sciences and Engineering Research Council of Canada grant (NSERC; Grant: RA4981A01), a Lawson Internal Research Fund Award (Lawson IRF), and a Canada Research Chair Tier 2 (CRC; Grant: 950-230372) to PM as well as a Canada Excellence Research Chair (CERC; Grant: 215063) award to AO.

## Conflict of Interest

The authors declare that the research was conducted in the absence of any commercial or financial relationships that could be construed as a potential conflict of interest.

## References

[1] DSM5 Diagnostic and Statistical Manual of Mental Disorders. 5th Edition (Arlington, VA: American Psychiatric Association) (2013).

[2] Sasson Y. Zohar J. Chopra M. Lustig M. Iancu I. Hendler T. Epidemiology of obsessive-compulsive disorder: a world view. J Clin Psychiatry (1997) 58, 7–10. 10.1159/000061358 9393390

[3] Bokor G Anderson PD Obsessive-compulsive disorder. J Pharm Pract (2014) 27:116–30. 10.1177/0897190014521996 24576790

[4] Mataix-Cols D Wooderson S Lawrence N Brammer MJ Speckens A Phillips ML DIstinct neural correlates of washing, checking, and hoarding symptomdimensions in obsessive-compulsive disorder. Arch Gen Psychiatry (2004) 61:564–76. 10.1001/archpsyc.61.6.564 15184236

[5] Torres AR Fontenelle LF Shavitt RG Ferrão YA do Rosário MC Storch EA Comorbidity variation in patients with obsessive–compulsive disorder according to symptom dimensions: results from a large multicentre clinical sample. J Affect Disord (2015) 190:508–16. 10.1016/j.jad.2015.10.051 26561941

[6] Packard MG Knowlton BJ Learning and memory functions of the Basal Ganglia. Annu Rev Neurosci (2002) 25:563–93. 10.1146/annurev.neuro.25.112701.142937 12052921

[7] Reading PJ Dunnett SB Robbins TW Dissociable roles of the ventral, medial and lateral striatum on the acquisition and performance of a complex visual stimulus-response habit. Behav Brain Res (1991) 45:147–61. 10.1016/S0166-4328(05)80080-4 1789923

[8] Burguiere E Monteiro P Mallet L Feng G Graybiel AM Striatal circuits, habits, and implications for obsessive-compulsive disorder. Curr Opin Neurobiol (2015) 30:59–65. 10.1016/j.conb.2014.08.008 25241072 PMC4293232

[9] Gotham AM Brown RG Marsden CD ‘frontal' cognitive function in patients with Parkinson's Disease ‘On' and ‘Off' Levodopa. Brain (1988) 111:299–321. 10.1093/brain/111.2.299 3378138

[10] MacDonald PA Monchi O Differential effects of dopaminergic therapies on dorsal and ventral striatum in Parkinson's disease: implications for cognitive function. Parkinsons Dis (2011) 2011:1–18. 10.4061/2011/572743 PMC3062097 21437185

[11] Feekes JA Cassell MD The vascular supply of the functional compartments of the human striatum. Brain (2006) 129:2189–201. 10.1093/brain/awl158 16815876

[12] Kish SJ Shannak K Hornykiewicz O Uneven pattern of dopamine loss in the striatum of patients with idiopathic Parkinson's Disease. New Engl J Med (1988) 318:876–80. 10.1056/NEJM198804073181402 3352672

[13] Tziortzi AC Haber SN Searle GE Tsoumpas C Long CJ Shotbolt P Connectivity-based functional analysis of dopamine release in the striatum using diffusion-weighted MRI and positron emission tomography. Cereb. Cortex (2014) 24:1165–77. 10.1093/cercor/bhs397 PMC3977617 23283687

[14] Hiebert NM Owen AM Seergobin KN MacDonald PA Dorsal striatum mediates deliberate decision making, not late-stage, stimulus-response learning. Hum Brain Mapp. (2017) 38:6133–56. 10.1002/hbm.23817 PMC6867067 28945307

[15] Robertson BD Hiebert NM Seergobin KN Owen AM MacDonald PA Dorsal striatum mediates cognitive control, not cognitive effort per se, in decision-making: An event-related fMRI study. Neuroimage (2015) 114:170–84. 10.1016/j.neuroimage.2015.03.082 25862263

[16] Yang XQ Glizer D Vo A Seergobin KN MacDonald PA Pramipexole increases go timeouts but not no-go errors in healthy volunteers. Front Hum Neurosci (2016) 10:523. 10.3389/fnhum.2016.00523 27803657 PMC5067488

[17] Atallah HE Lopez-Paniagua D Rudy JW O'Reilly RC Separate neural substrates for skill learning and performance in the ventral and dorsal striatum. Nat Neurosci (2007) 10:126–31. 10.1038/nn1817 17187065

[18] Hampshire A Hellyer PJ Parkin B Hiebert N MacDonald P Owen AM Network mechanisms of intentional learning. Neuroimage (2016) 127:123–34. 10.1016/j.neuroimage.2015.11.060 PMC4758826 26658925

[19] Hiebert NM Vo A Hampshire A Owen AM Seergobin KN MacDonald PA Striatum in stimulus-response learning via feedback and in decision making. Neuroimage (2014) 101:448–57. 10.1016/j.neuroimage.2014.07.013 25038436

[20] MacDonald PA MacDonald AA Seergobin KN Tamjeedi R Ganjavi H Provost JS The effect of dopamine therapy on ventral and dorsal striatum-mediated cognition in Parkinson's disease: support from functional MRI. Brain (2011) 134:1447–63. 10.1093/brain/awr075 21596772

[21] Piras F Piras F Chiapponi C Girardi P Caltagirone C Spalletta G Widespread structural brain changes in OCD: a systematic review of voxel-based morphometry studies. Cortex (2015) 62:89–108. 10.1016/j.cortex.2013.01.016 23582297

[22] Riffkin J Yucel M Maruff P Wood SJ Soulsby B Olver J A manual and automated MRI study of anterior cingulate and orbito-frontal cortices, and caudate nucleus in obsessive-compulsive disorder: comparison with healthy controls and patients with schizophrenia. Psychiatry Res (2005) 138:99–113. 10.1016/j.pscychresns.2004.11.007 15766634

[23] Carlisi CO Norman LJ Lukito SS Radua J Mataix-Cols D Rubia K Comparative multimodal meta-analysis of structural and functional brain abnormalities in autism spectrum disorder and obsessive-compulsive disorder. Biol Psychiatry (2017) 82:83–102. 10.1016/j.biopsych.2016.10.006 27887721

[24] Pujol J Soriano-Mas C Alonso P Cardoner N Menchon JM Deus J Mapping structural brain alterations in obsessive-compulsive disorder. Arch Gen Psychiatry (2004) 61:720–30. 10.1001/archpsyc.61.7.720 15237084

[25] Radua J Mataix-Cols D Voxel-wise meta-analysis of grey matter changes in obsessive-compulsive disorder. Br J Psychiatry (2009) 195:393–402. 10.1192/bjp.bp.108.055046 19880927

[26] Yoo SY Roh MS Choi JS Kang DH Ha TH Lee JM Voxel-based morphometry study of gray matter abnormalities in obsessive-compulsive disorder. J Korean Med Sci (2008) 23:24–30. 10.3346/jkms.2008.23.1.24 18303194 PMC2526479

[27] Zarei M Mataix-Cols D Heyman I Hough M Doherty J Burge L Changes in gray matter volume and white matter microstructure in adolescents with obsessive-compulsive disorder. Biol Psychiatry (2011) 70:1083–90. 10.1016/j.biopsych.2011.06.032 21903200

[28] Boedhoe PS Schmaal L Abe Y Ameis SH Arnold PD Batistuzzo MC Distinct subcortical volume alterations in pediatric and adult OCD: a worldwide meta- and mega-analysis. Am J Psychiatry (2017) 174:60–9. 10.1176/appi.ajp.2016.16020201 PMC5344782 27609241

[29] Kong XZ Boedhoe PSW Abe Y Alonso P Ameis SH Arnold PD Mapping cortical and subcortical asymmetry in obsessive-compulsive disorder: findings from the ENIGMA consortium. Biol Psychiatry (2019) 19:31292–2. 10.1016/j.biopsych.2019.04.022 PMC7094802 31178097

[30] Baxter Jr LR Phelps ME Mazziotta JC Guze BH Schwartz JM Selin CE Local cerebral glucose metabolic rates in obsessive-compulsive disorder: a comparison with rates in unipolar depression and in normal controls. Arch Gen Psychiatry (1987) 44:211–8. 10.1001/archpsyc.1987.01800150017003 3493749

[31] de Vries FE de Wit SJ van den Heuvel OA Veltman DJ Cath DC van Balkom A Cognitive control networks in OCD: a resting-state connectivity study in unmedicated patients with obsessive-compulsive disorder and their unaffected relatives. World J Biol Psychiatry (2017) 1–13. 10.1080/15622975.2017.1353132 28918693

[32] Del Casale A Kotzalidis GD Rapinesi C Serata D Ambrosi E Simonetti A Functional neuroimaging in obsessive-compulsive disorder. Neuropsychobiology (2011) 64:61–85. 10.1159/000325223 21701225

[33] Gursel DA Avram M Sorg C Brandl F Koch K Frontoparietal areas link impairments of large-scale intrinsic brain networks with aberrant fronto-striatal interactions in OCD: a meta-analysis of resting-state functional connectivity. Neurosci Biobehav Rev (2018) 87:151–60. 10.1016/j.neubiorev.2018.01.016 29410103

[34] Le Jeune F Verin M N'Diaye K Drapier D Leray E Du Montcel ST Decrease of prefrontal metabolism after subthalamic stimulation in obsessive-compulsive disorder: a positron emission tomography study. Biol Psychiatry (2010) 68:1016–22. 10.1016/j.biopsych.2010.06.033 20951978

[35] Perani D Colombo C Bressi S Bonfanti A Grassi F Scarone S [18F]FDG PET study in obsessive-compulsive disorder. A clinical/metabolic correlation study after treatment. Br J Psychiatry (1995) 166:244–50. 10.1192/bjp.166.2.244 7728370

[36] Rauch SL Neuroimaging and neuropsychology of the striatum. Bridging basic science and clinical practice. Psychiatr Clinics North America (1997) 20:741–68. 10.1016/S0193-953X(05)70343-9 9443348

[37] Remijnse PL Nielen MA van Balkom AM Cath DC van Oppen P Uylings HBM REduced orbitofrontal-striatal activity on a reversal learning task in obsessive-compulsive disorder. Arch Gen Psychiatry (2006) 63:1225–36. 10.1001/archpsyc.63.11.1225 17088503

[38] Nielen MM den Boer JA Smid HG Patients with obsessive-compulsive disorder are impaired in associative learning based on external feedback. Psychol Med (2009) 39:1519–26. 10.1017/S0033291709005297 19243647

[39] Norman LJ Taylor SF Liu Y Radua J Chye Y De Wit SJ Error processing and inhibitory control in obsessive-compulsive disorder: a meta-analysis using statistical parametric maps. Biol Psychiatry (2019) 85:713–25. 10.1016/j.biopsych.2018.11.010 PMC6474799 30595231

[40] Rubin RT Villanueva-Meyer J Ananth J Trajmar PG Mena I Regional xenon 133 cerebral blood flow and cerebral technetium 99m hmpao uptake in unmedicated patients with obsessive-compulsive disorder and matched normal control subjects: determination by high-resolution single-photon emission computed tomography. Arch Gen Psychiatry (1992) 49:695–702. 10.1001/archpsyc.1992.01820090023004 1514874

[41] Endrass T Kloft L Kaufmann C Kathmann N Approach and avoidance learning in obsessive-compulsive disorder. Depress Anxiety (2011) 28:166–72. 10.1002/da.20772 21284070

[42] Nakao T Nakagawa A Yoshiura T Nakatani E Nabeyama M Yoshizato C A functional MRI comparison of patients with obsessive-compulsive disorder and normal controls during a Chinese character Stroop task. Psychiatry Res (2005) 139:101–14. 10.1016/j.pscychresns.2004.12.004 15970434

[43] Hiebert NM Owen AM Ganjavi H Mendonca D Jenkins ME Seergobin KN Dorsal striatum does not mediate feedback-based, stimulus-response learning: an event-related fMRI study in patients with Parkinson's disease tested on and off dopaminergic therapy. Neuroimage (2018) 185:455–70. 10.1016/j.neuroimage.2018.10.045 30394326

[44] Macpherson T Morita M Hikida T Striatal direct and indirect pathways control decision-making behavior. Front Psychol (2014) 5:1301. 10.3389/fpsyg.2014.01301 25429278 PMC4228842

[45] Melief EJ McKinley JW Lam JY Whiteley NM Gibson AW Neumaier JF Loss of glutamate signaling from the thalamus to dorsal striatum impairs motor function and slows the execution of learned behaviors. npj. Parkinsons Dis, 4 (2018). 10.1038/s41531-018-0060-6 PMC6072777 30083593

[46] Beigi M Wilkinson L Gobet F Parton A Jahanshahi M Levodopa medication improves incidental sequence learning in Parkinson's disease. Neuropsychologia (2016) 93:53–60. 10.1016/j.neuropsychologia.2016.09.019 27686948 PMC5155668

[47] Helie S Roeder JL Ashby FG Evidence for cortical automaticity in rule-based categorization. J Neurosci (2010) 30:14225–34. 10.1523/JNEUROSCI.2393-10.2010 PMC6634766 20962243

[48] Kwak Y Müller MLTM Bohnen NI Dayalu P Seidler RD l-DOPA changes ventral striatum recruitment during motor sequence learning in Parkinson's disease. Behav Brain Res (2012) 230:116–24. 10.1016/j.bbr.2012.02.006 22343069

[49] MacDonald AA Monchi O Seergobin KN Ganjavi H Tamjeedi R MacDonald PA Parkinson's disease duration determines effect of dopaminergic therapy on ventral striatum function. Mov. Disord (2013a) 28:153–60. 10.1002/mds.25152 23165957

[50] MacDonald AA Seergobin KN Owen AM Tamjeedi R Monchi O Ganjavi H Differential effects of Parkinson's disease and dopamine replacement on memory encoding and retrieval. PloS One (2013b) 8:e74044. 10.1371/journal.pone.0074044 24086309 PMC3784427

[51] Postuma RB Dagher A Basal ganglia functional connectivity based on a meta-analysis of 126 positron emission tomography and functional magnetic resonance imaging publications. Cereb. Cortex (2006) 16:1508–21. 10.1093/cercor/bhj088 16373457

[52] Brett M Anton JL Valabregue V Poline JB Region of interest analysis using an SPM toolbox. In: Presented at the Eighth International Conference on Functional Mapping of the Human Brain. Sendai, Japan (2002).

[53] Dienes Z Using Bayes to get the most out of non-significant results. Front Psychol (2014) 5:781. 10.3389/fpsyg.2014.00781 25120503 PMC4114196

[54] Goodman WK Price LH Rasmussen SA The yale-brown obsessive compulsive scale: I. development, use, and reliability. Arch Gen Psychiatry (1989) 46:1006–11. 10.1001/archpsyc.1989.01810110048007 2684084

[55] Dhyani M Trivedi JK Nischal A Sinha PK Verma S Suicidal behaviour of Indian patients with obsessive compulsive disorder. Indian J Psychiatry (2013) 55:161–6. 10.4103/0019-5545.111455 PMC3696240 23825851

[56] Storch EA De Nadai AS Conceicao do Rosario M Shavitt RG Torres AR Ferrao YA Defining clinical severity in adults with obsessive-compulsive disorder. Compr Psychiatry (2015) 63:30–5. 10.1016/j.comppsych.2015.08.007 PMC4643407 26555489

[57] Figee M Vink M de Geus F Vulink N Veltman DJ Westenberg H Dysfunctional reward circuitry in obsessive-compulsive disorder. Biol Psychiatry (2011) 69:867–74. 10.1016/j.biopsych.2010.12.003 21272861

[58] Ali N Green DW Kherif F Devlin JT Price CJ The role of the left head of caudate in suppressing irrelevant words. J Cogn Neurosci (2010) 22:2369–86. 10.1162/jocn.2009.21352 PMC3646394 19803688

[59] Coderre E van Heuven W Modulations of the executive control network by stimulus onset asynchrony in a Stroop task. BMC Neurosci (2013) 14:79. 10.1186/1471-2202-14-79 23902451 PMC3734141

[60] Djamshidian A O'Sullivan SS Lees A Averbeck BB Stroop test performance in impulsive and non impulsive patients with Parkinson's disease. Parkinsonism Relat Disord (2011) 17:212–4. 10.1016/j.parkreldis.2010.12.014 PMC3042030 21247790

[61] Fera F Nicoletti G Cerasa A Romeo N Gallo O Gioia MC Dopaminergic modulation of cognitive interference after pharmacological washout in Parkinson's disease. Brain Res Bull (2007) 74:75–83. 10.1016/j.brainresbull.2007.05.009 17683792

[62] Larson MJ Clayson PE Primosch M Leyton M Steffensen SC The effects of acute dopamine precursor depletion on the cognitive control functions of performance monitoring and conflict processing: an event-related potential (ERP) Study. PloS One (2015) 10:e0140770. 10.1371/journal.pone.0140770 26492082 PMC4619587

[63] MacLeod CM Half a century of research on the stroop effect: an integrative review. psychol Bull (1991) 109:163–203. 10.1037/0033-2909.109.2.163 2034749

[64] MacLeod CM MacDonald PA Interdimensional interference in the Stroop effect: uncovering the cognitive and neural anatomy of attention. Trends In Cogn Sci (2000) 4:383–91. 10.1016/S1364-6613(00)01530-8 11025281

[65] Wright BC Wanley A Adults' versus children's performance in the Stroop task: Interference and facilitation. Br psychol Soc (2003) 94:475–85. 10.1348/000712603322503042 14687456

[66] Vriend C de Wit SJ Remijnse PL van Balkom AJ Veltman DJ van den Heuvel OA Switch the itch: a naturalistic follow-up study on the neural correlates of cognitive flexibility in obsessive-compulsive disorder. Psychiatry Res (2013) 213:31–8. 10.1016/j.pscychresns.2012.12.006 23693090

[67] van Velzen LS Vriend C de Wit SJ van den Heuvel OA Response inhibition and interference control in obsessive-compulsive spectrum disorders. Front Hum Neurosci (2014) 8:419. 10.3389/fnhum.2014.00419 24966828 PMC4052433

[68] Cavedini P Zorzi C Piccinni M Cavallini MC Bellodi L Executive dysfunctions in obsessive-compulsive patients and unaffected relatives: searching for a new intermediate phenotype. Biol Psychiatry (2010) 67:1178–84. 10.1016/j.biopsych.2010.02.012 20381015

[69] Grassi G Pallanti S Righi L Figee M Mantione M Denys D Think twice: impulsivity and decision making in obsessive-compulsive disorder. J Behav Addict (2015) 4:263–72. 10.1556/2006.4.2015.039 PMC4712760 26690621

[70] Rocha F. F. d. Alvarenga NB Malloy-Diniz L Corrêa H Decision-making impairment in obsessive-compulsive disorder as measured by the Iowa Gambling Task. Arquivos Neuro-Psiquiatria (2011) 69:642–7. 10.1590/S0004-282X2011000500013 21877034

[71] Evens R Hoefler M Biber K Lueken U The Iowa gambling task in parkinson's disease: a meta-analysis on effects of disease and medication. Neuropsychologia (2016) 91:163–72. 10.1016/j.neuropsychologia.2016.07.032 27475264

[72] Starcke K Tuschen-Caffier B Markowitsch HJ Brand M Dissociation of decisions in ambiguous and risky situations in obsessive-compulsive disorder. Psychiatry Res (2010) 175:114–20. 10.1016/j.psychres.2008.10.022 20004479

[73] Zhang L Dong Y Ji Y Zhu C Yu F Ma H Dissociation of decision making under ambiguity and decision making under risk: a neurocognitive endophenotype candidate for obsessive-compulsive disorder. Prog Neuropsychopharmacol Biol Psychiatry (2015) 57:60–8. 10.1016/j.pnpbp.2014.09.005 25315855

[74] Gillan CM Apergis-Schoute AM Morein-Zamir S Urcelay GP Sule A Fineberg NA Functional neuroimaging of avoidance habits in obsessive-compulsive disorder. Am J Psychiatry (2015) 172:284–93. 10.1176/appi.ajp.2014.14040525 PMC4910868 25526600

[75] Gillan CM Papmeyer M Morein-Zamir S Sahakian BJ Fineberg NA Robbins TW Disruption in the balance between goal-directed behavior and habit learning in obsessive-compulsive disorder. Am J Psychiatry (2011) 168:718–26. 10.1176/appi.ajp.2011.10071062 PMC3533260 21572165

[76] Gillan CM Robbins TW Sahakian BJ van den Heuvel OA van Wingen G The role of habit in compulsivity. Eur Neuropsychopharmacol (2016) 26:828–40. 10.1016/j.euroneuro.2015.12.033 PMC4894125 26774661

[77] Marton T Samuels J Nestadt P Krasnow J Wang Y Shuler M Validating a dimension of doubt in decision-making: a proposed endophenotype for obsessive-compulsive disorder. PloS One (2019) 14:e0218182. 10.1371/journal.pone.0218182 31194808 PMC6564001

[78] Sarig S Dar R Liberman N Obsessive-compulsive tendencies are related to indecisiveness and reliance on feedback in a neutral color judgment task. J Behav Ther Exp Psychiatry (2012) 43:692–7. 10.1016/j.jbtep.2011.09.012 21983353

[79] Stern ER Welsh RC Gonzalez R Fitzgerald KD Abelson JL Taylor SF Subjective uncertainty and limbic hyperactivation in obsessive-compulsive disorder. Hum Brain Mapp (2013) 34:1956–70. 10.1002/hbm.22038 PMC5289818 22461182

[80] Aarts E Roelofs A Franke B Rijpkema M Fernandez G Helmich RC Striatal dopamine mediates the interface between motivational and cognitive control in humans: evidence from genetic imaging. Neuropsychopharmacology (2010) 35:1943–51. 10.1038/npp.2010.68 PMC3055632 20463658

[81] Jung WH Kang DH Han JY Jang JH Gu BM Choi JS Aberrant ventral striatal responses during incentive processing in unmedicated patients with obsessive-compulsive disorder. Acta Psychiatr Scand (2011) 123:376–86. 10.1111/j.1600-0447.2010.01659.x 21175552

[82] Tachibana Y Hikosaka O The primate ventral pallidum encodes expected reward value and regulates motor action. Neuron (2012) 76:826–37. 10.1016/j.neuron.2012.09.030 PMC3519929 23177966

[83] Menzies L Chamberlain SR Laird AR Thelen SM Sahakian BJ Bullmore ET Integrating evidence from neuroimaging and neuropsychological studies of obsessive-compulsive disorder: the orbitofronto-striatal model revisited. Neurosci Biobehav Rev (2008) 32:525–49. 10.1016/j.neubiorev.2007.09.005 PMC2889493 18061263

[84] Rauch SL Jenike MA Alpert NM Baer L Breiter HCR Savage CR Regional cerebral blood flow measured during symptom provocation in obsessive-compulsive disorder using oxygen 15—labeled carbon dioxide and positron emission tomography. Arch Gen Psychiatry (1994) 51:62–70. 10.1001/archpsyc.1994.03950010062008 8279930

[85] Figee M Pattij T Willuhn I Luigjes J van den Brink W Goudriaan A Compulsivity in obsessive-compulsive disorder and addictions. Eur Neuropsychopharmacol (2016) 26:856–68. 10.1016/j.euroneuro.2015.12.003 26774279

